# Loss of MYSM1 inhibits the oncogenic activity of cMYC in B cell lymphoma

**DOI:** 10.1111/jcmm.16554

**Published:** 2021-06-11

**Authors:** Yun Hsiao Lin, HanChen Wang, Amanda Fiore, Michael Förster, Lin Tze Tung, Jad I Belle, Francis Robert, Jerry Pelletier, David Langlais, Anastasia Nijnik

**Affiliations:** ^1^ Department of Physiology McGill University Montreal QC Canada; ^2^ McGill University Research Centre on Complex Traits McGill University QC Canada; ^3^ Department of Human Genetics McGill University Montreal QC Canada; ^4^ Department of Biochemistry McGill University Montreal QC Canada; ^5^ The Rosalind and Morris Goodman Cancer Research Centre McGill University Montreal QC Canada; ^6^ McGill University Genome Centre McGill University Montreal QC Canada; ^7^ Department of Microbiology and Immunology McGill University Montreal QC Canada

**Keywords:** B cell lymphoma, chromatin, cMYC, mouse models, transcriptional regulation

## Abstract

MYSM1 is a chromatin‐binding protein, widely investigated for its functions in haematopoiesis in human and mouse; however, its role in haematologic malignancies remains unexplored. Here, we investigate the cross‐talk between MYSM1 and oncogenic cMYC in the transcriptional regulation of genes encoding ribosomal proteins, and the implications of these mechanisms for cMYC‐driven carcinogenesis. We demonstrate that in cMYC‐driven B cell lymphoma in mouse models, MYSM1‐loss represses ribosomal protein gene expression and protein synthesis. Importantly, the loss of MYSM1 also strongly inhibits cMYC oncogenic activity and protects against B cell lymphoma onset and progression in the mouse models. This advances the understanding of the molecular and transcriptional mechanisms of lymphomagenesis, and suggests MYSM1 as a possible drug target for cMYC‐driven malignancies.

## 1 INTRODUCTION

cMYC is a transcription factor that stimulates ribosome production, protein synthesis, cell growth and proliferation, and many other cellular functions.^1^ cMYC is also a highly potent oncogene, over‐expressed, amplified or otherwise deregulated in over 50% of all cancers.^2^ Among haematologic cancers, the *cMYC* locus is commonly rearranged or amplified in non‐Hodgkin B cell lymphomas, including ~80% of Burkitt lymphomas and ~10% of diffuse large B cell lymphomas (DLBCL).^3^ In particular in DLBCL, *cMYC* chromosomal translocations are associated with rapid disease progression and poor response to therapy.^3^, ^4^ New strategies for the treatment of cMYC‐driven lymphoid malignancies are therefore urgently needed.

Cancers with *cMYC*‐aberrations require continued cMYC expression and function to persist and progress, and this makes cMYC an attractive drug‐target.^4^ However, the lack of ligand‐binding domains or catalytic activity make direct repression of cMYC highly challenging.^4^, ^5^, ^6^ Most drugs in development aim to inhibit cMYC activity indirectly by targeting other proteins that interact with and regulate cMYC.^6^ Importantly, the oncogenic activity of cMYC has been directly linked to its roles in the stimulation of ribosome production and protein synthesis.^1^, ^7^, ^8^

MYSM1 is a chromatin‐binding protein with deubiquitinase catalytic activity (DUB).^9^ One of the main known MYSM1 substrates is histone H2A, monoubiquitinated at K119, and MYSM1 catalytic activity on this epigenetic mark promotes the activation of gene expression.^9^ We recently conducted the first genome‐wide analysis of MYSM1‐regulated genes and demonstrated that in primary murine haematopoietic stem and progenitor cells MYSM1 promotes the expression of many genes encoding ribosomal proteins and translation factors.^10^ Interestingly, MYSM1 was previously shown to interact with cMYC in B1a lymphocytes^11^ ; however, the role of MYSM1 as a transcriptional regulator in haematologic malignancies remains unexplored. As cMYC is the major transcriptional regulator for the genes encoding the ribosomal and translational machinery, here we investigate the cross‐talk between MYSM1 and cMYC in the regulation of these gene‐sets and its implications for cMYC‐driven carcinogenesis. Overall, we demonstrate that the loss of MYSM1 in mouse B cell lymphoma represses the induction of ribosomal protein genes, reduces cellular protein synthesis rate, promotes p53 activation and potently inhibits cMYC oncogenic functions.

**FIGURE 1:**
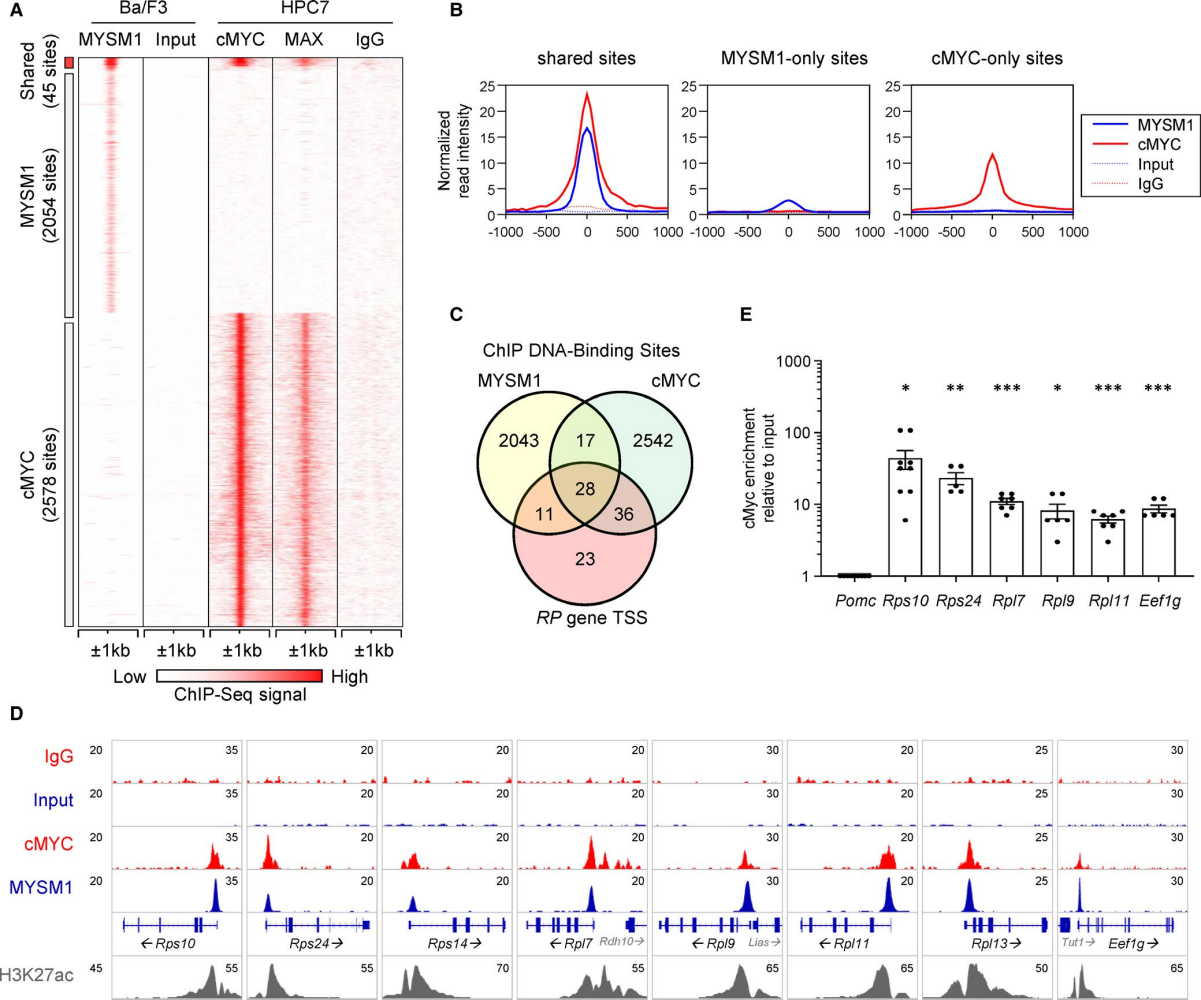
Co‐localization of MYSM1 and cMYC DNA‐binding sites at the promoters of genes encoding ribosomal proteins (*RPs*). A, Consolidation of genome‐wide DNA‐binding data for MYSM1, cMYC and MAX using ChIP‐seq datasets from Belle et al, *JCI Insight* 2020 (MYSM1)^10^ and Wilson et.al., *Blood* 2016 (cMYC, MAX),^13^ highlighting 45 shared binding sites between MYSM1 and cMYC/MAX. Input and IgG served as background controls for the two cell lines. B, Graphs showing the average normalized read intensities of MYSM1 and cMYC around the shared, MYSM1‐only, and cMYC‐only binding sites, from the ChIP‐seq datasets above. C, A Venn diagram comparing MYSM1 and cMYC DNA‐binding sites, and showing the number of binding sites within 1kb of a transcription start site (TSS) of a ribosomal protein gene (*RP* gene). This demonstrates that at least 28 *RP* gene promoters carry both MYSM1 and cMYC binding sites; please note that we do not exclude that MYSM1 and cMYC may also regulate other *RP* genes, not detected in these ChIP‐seq datasets due to inherent limitations of the ChIP method. D, Genomic snapshots of cMYC and MYSM1 binding near select *RP* gene promoters. Data for MYSM1 are from Belle et al, *JCI Insight* 2020^10^ ; data for cMYC are from Wilson et.al., *Blood* 2016.^13^ E, Validation of cMYC binding at known MYSM1 DNA‐binding sites at the promoters of genes encoding ribosomal proteins and translation factors, in a B cell precursor cell line Ba/F3 using ChIP‐qPCR. All Ct values were normalized to those of the pro‐opiomelanocortin (*Pomc*) gene, which serves as a negative binding region. Enrichment was calculated relative to input DNA. A one sample t test was performed, testing whether each dataset mean is different from ‘1’, corresponding to the signal at the negative control region *Pomc*, to indicate significant enrichment of cMYC at that genomic location; *P < .05, **P < .01, ***P < .001

To compare the location of the genomic binding sites of MYSM1 and cMYC, we consolidated the ChIP‐Seq datasets for cMYC and its dimerization partner MAX from multipotent haematopoietic progenitor cells HPC7 ^12^, ^13^ with the MYSM1 ChIP‐Seq acquired in our recent work in a B cell progenitor cell line Ba/F3.^10^ This identified 45 binding sites shared by MYSM1 and cMYC (Figure 1A‐B), all located within 1kb to the nearest gene transcription start site (TSS, Table S1). Importantly, 28 of these shared binding sites localized near the genes encoding ribosomal proteins (RPs) and 4 others near the genes encoding translation factors (Figure 1C‐D, Table S1). Overall, this suggested a possible cooperation between cMYC and MYSM1 in the transcriptional regulation of genes encoding ribosomal proteins and translation factors.

We have previously validated MYSM1 binding to the promoters of *RP* genes in Ba/F3 cells by ChIP‐qPCR, and also demonstrated a reduction in *RP*‐gene expression in *Mysm1*‐shRNA knockdown Ba/F3 cells and in *Mysm1*‐deficient primary haematopoietic stem and progenitor cells.^10^ To further validate the overlap in the genomic binding sites of MYSM1 and cMYC in Ba/F3 cells, the binding of cMYC at the MYSM1‐binding sites of select genes encoding RPs and translation factors was tested and confirmed with ChIP‐qPCR (Figure 1E, Supporting Information S1). The binding of MYSM1 and cMYC at the shared sites was also confirmed in cells derived from *EuMyc* mouse B cell lymphoma,^14^, ^15^ specifically for the *Rpl7* and *Eef1g* gene promoters (data not shown). We further assessed the effect of MYSM1 knockdown on cMYC binding, with ChIP‐qPCR analyses comparing *Mysm1*‐shRNA knockdown and control Ba/F3 cells.^10^ We observed no significant effect of MYSM1 knockdown on cMYC binding at select *RP* gene promoters (data not shown). Overall, we demonstrate shared DNA binding of cMYC and MYSM1 at the promoters of genes encoding ribosomal proteins and translation factors. Our data also suggest that MYSM1 maintains *RP*‐gene expression not by facilitating cMYC recruitment, but likely by other molecular mechanisms.

**FIGURE 2:**
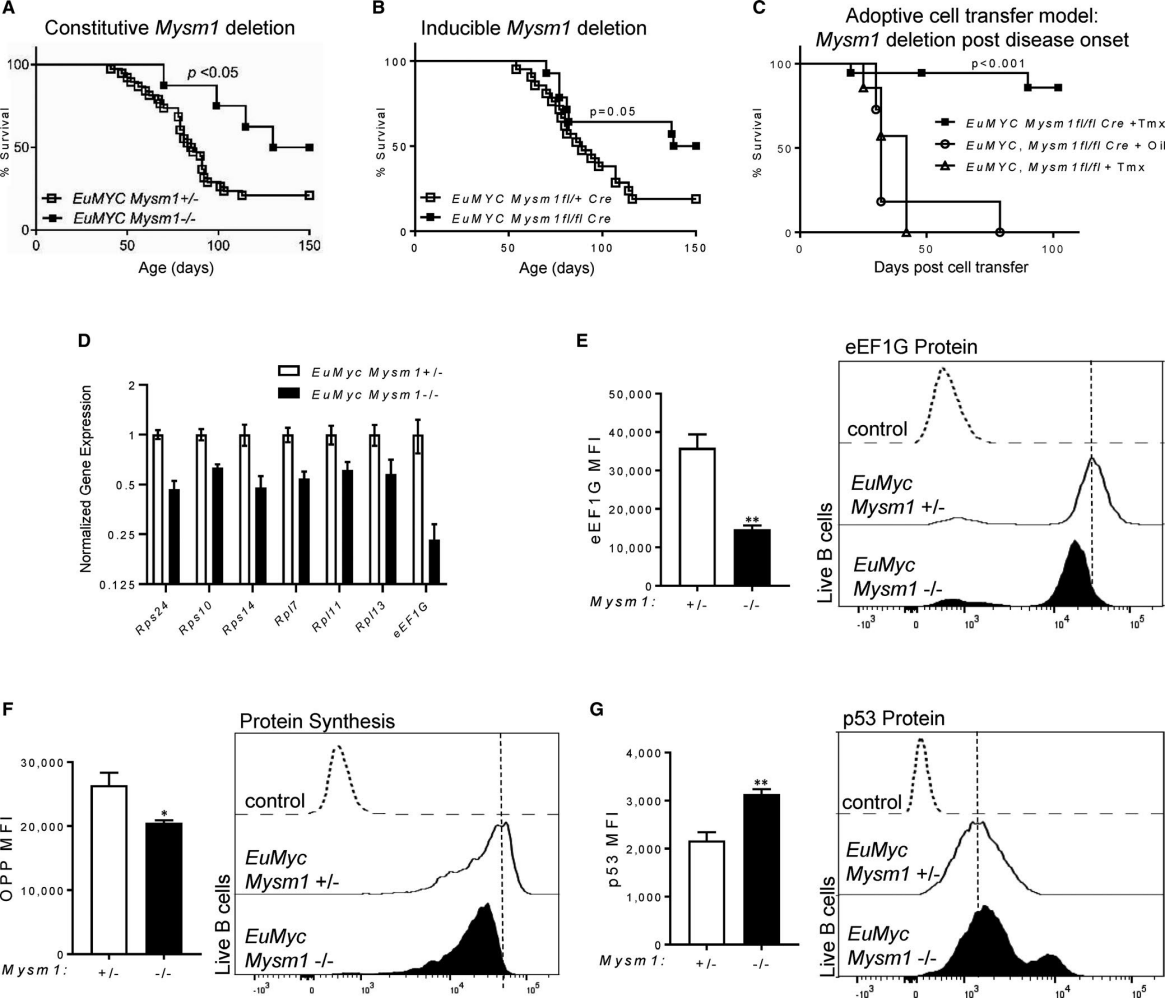
Loss of MYSM1 protects against B cell lymphoma onset and progression in mouse models, via the reduction in expression of the genes encoding ribosomal and protein translation machinery. A, Survival of *EuMyc Mysm1^‐/‐^* (n = 8) relative to control *EuMyc Mysm1^+/‐^* littermates (n = 38). B, Survival of tamoxifen‐treated *EuMyc Mysm1^fl/fl^ Cre^ERT2^* mice (n = 14), relative to tamoxifen‐treated *EuMyc Mysm1^fl/+^ Cre^ERT2^* control littermates (n = 21). Note that *Mysm1^+/‐^* and *Mysm1^fl/+^* were used as controls, as they were bred as littermates, age and sex matched, and maintained in the same cages as mice of the main experimental groups; mice lacking one *Mysm1* allele are known to be phenotypically equivalent to wild type, according to previous studies.^10^, ^16^ C, Survival of wild‐type recipient mice after adoptive transfer of 10^6^ *EuMyc Mysm1^fl/fl^ Cre^ERT2^* lymphoma cells followed by tamoxifen (Tmx) treatment (n = 18), relative to control recipient mice administered with the same number of *Cre^ERT2^*‐negative lymphoma cells followed by Tmx‐treatment (n = 7), and relative to control recipients receiving cells of the same genotype followed by vehicle corn oil (n = 11). *p*‐values are calculated using log‐rank (Mantel‐Cox) test. D‐G, Characterization of *EuMyc* primary lymphoma cells from *EuMyc Mysm1^‐^* ^/‐^ and control *EuMyc Mysm1^+/‐^* mice. D, Reduced expression of the genes encoding ribosomal proteins and the translation factor eEF1G in *Mysm1*‐deficient relative to control *EuMyc* lymphoma cells, measured by qRT‐PCR and normalized to *Hprt* and to the average expression in the *EuMyc Mysm1* ^+/‐^ control group. Live B220^+^ cells were FACS‐sorted from tumours for RNA isolation and qRT‐PCR analyses. E, Reduced levels of eEF1G translation initiation factor in the *Mysm1*‐deficient relative to control *EuMyc* cells measured by intracellular flow cytometry. F, Protein synthesis rates measured using OPP‐incorporation method and flow cytometry, showing a reduction in *Mysm1*‐deficient relative to control *EuMyc* lymphoma cells. G, Elevated levels of p53 protein in *Mysm1*‐deficient relative to control *EuMyc* lymphoma cells, measured with intracellular flow cytometry. In panels (E‐G), mean fluorescence intensity (MFI) of live B220^+^ lymphoma cells is plotted for each genotype and parameter studied, and representative flow cytometry histograms showing OPP incorporation, eEF1G levels and p53 levels in live B220^+^ lymphoma cells of each genotype are provided, with the control samples representing non‐specific background staining estimated with isotype control antibodies. Statistical analyses using Student's t test, * *P* <.05, ** *P* <.01

As the induction of the transcriptional programmes of ribosome biogenesis is critical for cMYC oncogenic activity,^1^, ^7^, ^8^ we hypothesized that MYSM1‐loss may interfere with cMYC oncogenic functions. This was tested in the *EuMyc* mouse model of B cell lymphoma that overexpresses cMYC under the control of the immunoglobulin heavy chain locus enhancer.^14^ The *EuMyc* mouse line was crossed to our established *Mysm1* ^‐/‐^ and *Mysm1* ^fl/fl^ *Cre^ERT2^* mouse lines, allowing either constitutive or tamoxifen‐induced *Mysm1*‐deletion.^16^, ^17^ Protective effects were seen with *Mysm1*‐deletion in both models, as demonstrated by the increased lifespan of *EuMyc Mysm1* ^‐/‐^ and tamoxifen‐treated *EuMyc Mysm1* ^fl/fl^ *Cre^ERT2^* mice relative to corresponding control *EuMyc* groups (Figure 2A‐B). This indicates that the loss of MYSM1 can inhibit the oncogenic activity of cMYC and delay the onset of fatal lymphoma.

To further establish the protective effect of MYSM1‐loss on lymphoma disease progression, we employed an adoptive lymphoma cell transfer model. Lymphoma cells were harvested from *EuMyc Mysm1* ^fl/fl^ *Cre^ERT2^* and control *EuMyc Mysm1* ^fl/fl^ donor mice, and transferred into independent cohorts of wild‐type C57BL/6 recipient mice at 10^6^ cells per mouse via intravenous injections. The recipient mice were treated either with tamoxifen to induce *Mysm1*‐deletion or with vehicle corn oil. Thereby, we demonstrated that *Mysm1*‐deletion had a striking protective activity, with strong extension in mouse survival and full remission in many of the treated animals (Figure 2C). This firmly establishes that loss of MYSM1 inhibits the oncogenic activity of cMYC, and protects against B cell lymphoma onset and progression in mouse models.

To understand the mechanisms underlying the protective activity of MYSM1‐loss in B cell lymphoma, *EuMyc* tumours were harvested from *Mysm1* ^‐/‐^ and control mice, and lymphoma cells isolated by cell‐sorting as live B220^+^ cells, for ex vivo analyses with qRT‐PCR and intracellular flow cytometry. We observed a significant down‐regulation in the expression of genes encoding ribosomal proteins and translation factors in *EuMyc Mysm1* ^‐/‐^ relative to control *EuMyc* lymphoma cells (Figure 2D). A reduction in the translation factor eEF1G in *EuMyc Mysm1* ^‐/‐^ lymphoma cells was further validated by flow cytometry at the protein level (Figure 2E). This was associated with a reduction in the overall protein synthesis rate in *EuMyc Mysm1* ^‐/‐^ relative to control *EuMyc* lymphoma cells, and an increase in the levels of the p53 tumour suppressor protein (Figure 2F‐G). Importantly, previous studies in the *EuMyc* mouse model have shown that ribosomal dysfunction can restrain cMYC oncogenic activity via both a reduction in cellular protein synthesis ^18^ and via the activation of p53.^19^ In future work, it will be important to address the relative contribution of these pathways to the protective effects of *Mysm1*‐deficiency in *EuMyc* B cell lymphoma, and this will provide insights into the possible effects of acquired p53 mutations on this protective activity. Overall, we establish that the loss of MYSM1 protects against B cell lymphoma onset and progression in the *EuMyc* mouse model via a reduction in the expression of genes encoding the ribosomal and translational machinery.

Taken together, our work demonstrates that MYSM1 is required to sustain the oncogenic activity of cMYC in B cell lymphoma, by promoting the cMYC‐dependent induction of the genes encoding ribosomal proteins and translation factors. This suggests MYSM1 as a potential drug target for B cell lymphoma and possibly other haematologic malignancies with *cMYC*‐locus rearrangements and amplifications. Future work will need to address whether the loss of MYSM1 DUB catalytic activity, rather than the complete loss of MYSM1 protein, exerts a similar protective effect, potentially providing a rationale for the development of MYSM1 inhibitors as novel chemotherapeutic agents. Drugs targeting other members of the zinc metalloproteinase family have entered clinical trials,^20^ suggesting that the development of MYSM1 small‐molecule antagonists for in vivo use may be feasible. Our data lend credence to the hypothesis that such compounds may repress cMYC‐driven expression of ribosomal and translational machinery, and may therefore synergistically enhance the efficacy of inhibitors that directly target the ribosome, in development for cancer chemotherapy.^21^, ^22^

## CONFLICTS OF INTEREST

The authors confirm that there are no conflicts of interest.

## AUTHOR CONTRIBUTIONS

**Yun Hsiao Lin:** Data curation (lead); Formal analysis (lead); Investigation (lead); Methodology (supporting); Validation (lead); Writing‐review & editing (supporting). **HanChen Wang:** Data curation (supporting); Formal analysis (supporting); Investigation (supporting); Software (lead); Writing‐review & editing (supporting). **Amanda Fiore:** Data curation (supporting); Formal analysis (supporting); Investigation (supporting); Writing‐review & editing (supporting). **Michael** **Förster:** Data curation (supporting); Formal analysis (supporting); Investigation (supporting); Supervision (supporting); Writing‐review & editing (supporting). **Lin** **Tze Tung:** Investigation (supporting); Writing‐review & editing (supporting). **Jad I. Belle:** Conceptualization (supporting); Supervision (supporting); Writing‐review & editing (supporting). **Francis Robert:** Methodology (supporting); Resources (supporting); Supervision (supporting); Writing‐review & editing (supporting). **Jerry Pelletier:** Methodology (supporting); Resources (supporting); Supervision (supporting). **David** **Langlais:** Methodology (supporting); Resources (supporting); Software (supporting); Supervision (supporting); Writing‐review & editing (supporting). **Anastasia** **Nijnik:** Conceptualization (lead); Funding acquisition (lead); Methodology (lead); Project administration (lead); Supervision (lead); Writing‐original draft (lead); Writing‐review & editing (lead).

## Supporting information

Table S1

Supporting Information S1

## Data Availability

Supporting data is included in Supplemental Materials. ChIP‐Seq data are available in the National Center for Biotechnology Information GEO database under the following accession number: GSE150667.
