# Supplementary material for: Loss of MYSM1 inhibits the oncogenic activity of cMYC in B cell lymphoma
Source: J Cell Mol Med. 2021 Jun 11;25(14):7089–94. doi: 10.1111/jcmm.16554 (PMC8278115; doi:10.1111/jcmm.16554)
Supplement: Supplementary file 2 — Supporting Information S1 [file JCMM-25-7089-s002.pdf]

SUPPLEMENTAL MATERIALS AND METHODS

Mouse Lines

Mouse line B6.Cg-Tg(IghMyc)22Bri/J, also known as *EuMyc*, is a widely studied model of B cell lymphoma and overexpresses cMYC under the control of the immunoglobulin heavy chain locus enhancer[1]. Mouse lines *Mysm1*$^{-/-}$ and *Mysm1*$^{fl/fl}$ carry the loss-of-function and the conditional alleles of *Mysm1* gene, respectively, and were previously described[2-4]. *Mysm1*$^{fl/fl}$*Cre*^{ERT2} mice were derived for tamoxifen-induced *Mysm1*-gene deletion by crossing the *Mysm1*$^{fl/fl}$ and Gt(ROSA)26Sor$^{tm1(cre/ERT2)}$ strains, as previously described[4]. All lines were on the C57BL/6 genetic background. The mice were maintained under specific pathogen-free conditions. All experiments were in accordance with the guidelines of the Canadian Council on Animal Care, and protocol AUP-7643 approved by the McGill University Animal Care Committee.

Tamoxifen Mouse Treatment

For tamoxifen-induced *Mysm1*-gene deletion, mice of *Mysm1*$^{fl/fl}$*Cre*^{ERT2} and control genotypes were injected intraperitoneally with tamoxifen (Sigma-Aldrich, T5648) in sterilized corn oil at 0.15 mg/gram per injection, with 8 doses administered in total over 16 days. Successful deletion of *Mysm1* exon 3 was validated by PCR analyses of the genomic DNA from hematopoietic and lymphoid organs of the mice, as described previously[4, 5]. Control animals of the same genotypes injected with vehicle corn oil (Sigma-Aldrich) were also included in the analyses.

Adoptive Transfer of B cell Lymphoma Cells

Tumour cells harvested from mice of *EuMyc Mysm1*$^{fl/fl}$*Cre*^{ERT2} and control *EuMyc Mysm1*$^{fl/fl}$ genotypes were processed to single cell suspension, subjected to red blood cell lysis in ACK buffer (0.15M NH4Cl, 10mM KHCO3, 0.1mM EDTA) and cryopreserved. Subsequently, the tumour cells were transferred via an intravenous injection at 10^6 cells per recipient, into wild type C57BL/6 mice, previously subjected to 3.5 Gy whole body irradiation in a RS2000 irradiator (Rad Source). The recipients were administered with tamoxifen or vehicle corn oil, as described above[4, 5], and mouse health and survival monitored over subsequent 100 days. Survival was defined as the time to the terminal stage of disease, at which point the animals were euthanized as determined in the protocol AUP-7643 approved by the McGill University Animal Care Committee.

Culture of Cell Lines

Murine B cell line Ba/F3 was maintained at 0.5-2 x10^6 cells/mL in RPMI-1640 (Wisent) with 10% Fetal Calf Serum (FCS;Wisent), 2mM L-Glutamine, 100μg/mL streptomycin, 100U/mL penicillin (Wisent), and 5% WEHI conditioned media as the source of IL-3. Ba/F3 cell line stably expressing triple-FLAG-tagged murine MYSM1 was previously described[5]. *EuMyc* lymphomas cells[6], expressing the same triple-FLAG-tagged murine MYSM1 construct, were derived through retroviral transduction with pMSCV vector (Addgene), as previously described[7]. The stable lines were maintained under 2μg/mL puromycin selection (Wisent).

Culture of Primary Cells

Tumour cells harvested from spleen and lymph nodes of *EuMyc* mice of different *Mysm1* genotypes were cultured on a monolayer of irradiated *Ink4a*$^{-/-}$ mouse embryonic fibroblasts (MEFs) in media containing 45% DMEM (Life Technologies), 45% IMEM (Life Technologies),

10% FCS (Wisent), 100μg/mL streptomycin and 100U/mL penicillin (Wisent), and $5x10^{-5}$ M β-mercaptoethanol (Sigma-Aldrich)[6].

Flow Cytometry and Cell Sorting

Cell suspensions of mouse spleen and lymph nodes were prepared in 45% DMEM (Life Technologies), 45% IMEM (Life Technologies), 10% FCS (Wisent), 100μg/ml streptomycin and 100U/ml penicillin (Wisent), and $5x10^{-5}$ M β-mercaptoethanol (Sigma-Aldrich). The cells were stained for surface-markers in PBS with 2% FCS for 20 minutes on ice with eFluor450-conjugated anti-CD45R/B220 (RA3–6B2, BioLegend). Fixable Viability Dye eFluor506 (eBioscience) was used to discriminate dead cells, and compensation done with BD™ CompBeads (BD Biosciences).

Intracellular staining for flow cytometry was performed as previously described[5, 8]. Briefly, the cells were fixed in 2% paraformaldehyde (PFA) in PBS with 2% FCS at 37℃ for 10 minutes, and permeabilized in 90% methanol for 30 minutes on ice. The cells were stained with intracellular antibodies: Alexa Fluor 488 anti-p53 (clone 1C12, Cell Signaling), or unconjugated anti-cMYC (clone D84C12, Cell Signaling) or anti-eEF1G (EPR7200, Abcam) with Alexa Fluor 488 anti-rabbit IgG highly cross-adsorbed secondary antibody (Life Technologies), or appropriate isotype controls. All data were acquired on FACS Canto II flow cytometer (BD Biosciences) and analyzed with FACS Diva (BD Biosciences) or FlowJo (Tree Star) software.

Cell sorting was performed on FACSAria II (BD Biosciences), with cells pre-stained with PE anti-IgM (II/41, eBioscience), PerCP-Cy5.5 anti-CD45R/B220 (RA3–6B2, BioLegend), and DAPI to discriminate dead cells.

Protein Synthesis Rate Measurements

Analysis of protein synthesis rates was performed using the O-propargyl-puromycin (OPP) incorporation method. Briefly, cells were cultured in the presence of 20 μM OPP for 30 minutes, stained with Fixable Viability Dye eFluor506 (eBioscience), fixed in 2% paraformaldehyde (PFA) in PBS with 2% FCS at 37℃ for 10 minutes, and permeabilized in 90% methanol for 30 minutes on ice. The cells were then washed with PBS, and staining for OPP incorporation using the Click-iT™ Plus OPP Alexa Fluor 488 Protein Synthesis Assay Kit (Life Technologies, Thermo Fisher Scientific) according to the manufacturer's protocols. Samples were analyzed by flow cytometry on FACS Canto II with FACS Diva software (BD Biosciences).

RNA Isolation and qPCR

RNA isolation from cell lines was carried out using the MagMAX total RNA kit (Ambion, Life Technologies) according to the manufacturer’s protocol. RNA quality was assessed on Bioanalyzer RNA Pico chips (Agilent), and cDNA was prepared using the qScript XLT cDNA Supermix (Quanta Biosciences) with 2-5ng RNA input per reaction. qPCRs were performed on a StepOnePlus instrument with Power SYBR Mastermix (Applied Biosystems, Life Technologies). The primers were purchased from IDT Technologies, and the sequences are provided in Supplemental Table S2.

Chromatin Immunoprecipitation

ChIP was performed as described previously[5, 9], with minor modifications. Briefly, cells were fixed with 1% formaldehyde in the culture media for 10 minutes at room temperature, followed by

addition of 0.125M of glycine to stop fixation. Nuclei were extracted with 5 minutes lysis in 0.25% Triton buffer (10mM Tris-HCl pH8, 10mM EDTA, 0.5mM EGTA), followed by 30 minutes in 200mM NaCl buffer (10mM Tris-HCl pH8, 1mM EDTA, 0.5mM EGTA). Nuclei were resuspended in sonication buffer (10mM Tris pH8, 140mM NaCl, 1mM EDTA, 0.5mM EGTA, 0.5% SDS, 0.5% Triton X-100, 0.05% NaDOC) and sonicated for twelve cycles of 30 seconds with a digital sonifier (Branson Ultrasonics at 80%, with 30 seconds rest in cooled circulating water.

Beads were prepared overnight with 40μL of Dynabeads Protein G (Invitrogen, Life Technologies) conjugated to 5μg of antibodies: anti-FLAG M2 (Sigma, F1804) or anti-cMYC (clone D84C12, Cell Signaling). Immunoprecipitation was performed by overnight incubation of antibody-bead matrices with sheared chromatin from the equivalent of $5x10^6$ cells. Immune complexes were washed sequentially for 2 min at room temperature with 1 ml of the following buffers: wash B (1% Triton X-100, 0.1% SDS, 150-mM NaCl, 2-mM EDTA, and 20-mM Tris-HCl, pH 8), wash C (1% Triton X-100, 0.1% SDS, 500-mM NaCl, 2-mM EDTA, and 20-mM Tris-HCl, pH 8), wash D (1% NP-40, 250-mM LiCl, 1-mM EDTA, and 10-mM Tris-HCl, pH 8), and TEN buffer (50-mM NaCl, 10-mM Tris-HCl, pH 8, and 1-mM EDTA). The samples were de-crosslinked by overnight incubation at 65℃ in 1% SDS buffer (50mM Tris-HCl pH8, 10mM EDTA). Following RNaseA and Proteinase K enzymatic treatments, ChIP DNA was purified using Qiaquick PCR Cleanup kit (Qiagen). ChIP enrichment was quantified using qPCR analysis, with primer sequences provided in Table S3. All C_T values were normalized to those of the pro-opiomelanocortin (*Pomc*) gene, which serves as a negative binding region. Enrichment was calculated relative to input DNA.

ChIP-Seq data analyses

The following ChIP-Seq datasets were included in the analyses: (I) cMYC ChIP-Seq from HPC7 murine hematopoeitic progenitor cells from Wilson NK et. al. [10, 11]; (II) MYSM1 ChIP-Seq from Ba/F3 murine B cell progenitors stably expressing 3xFLAG-tagged MYSM1 [12]. The reads were mapped to the UCSC mouse mm9 reference genome with Bowtie 1.0.0[13], and chromatin binding sites identified using peak detection algorithm MACS1.4.1[14], with comparisons for read enrichment against control input DNA from the same cells. Normalized sequence read density profiles (bigwig) were generated with Homer tool[15] and visualized with IGV[16].

Statistical analyses

Statistical comparisons were performed with Prism 7.01 (GraphPad), using Student's *t*-test for two groups, ANOVA for multiple comparisons, and Log-rank (Mantel-Cox) test for survival data.

Supplemental Table S2. RT-qPCR Primers Sequences.

Target Gene	Forward Sequence	Reverse Sequence
Rps3	ctgaaggcagcgtagagctt	tccaaggagtttgtagcgtaga
Rps10	gtgagcgacctgcaagattc	cagcctcagctttcttgtca
Rps24	gcagtgagcggtcctcttt	ggtccggatggttactgtgt
Rpl7	ccttgattgctcggtctctt	agcctgtttatctggtcttcc
Rpl9	catccaggagaatggctcttt	cagttcccttctcagacacatag
Rpl11	aaatgagaagattgctgttcactg	caactcatactcccgcacct
Rpl13	gaaacaagtccacggagtca	ttgctcggatgccaaaga
Eef1g	tcacgagaggagaaacagaaac	cagggaccagccatctttatc
Hprt	caggccagactttgttggat	ttgcgctcatcttaggcttt
Mysm1	gggattccgacctaacttgtc	tggaaaggaacagattttctatttg

Supplemental Table S3. ChIP-qPCR Primer Sequences.

Target Region (mm9)	Forward Sequence	Reverse Sequence
Rps3 98 bp downstream (Chr7:106,631,961-106,632,121)	aatacacaatctacggccatcc	agatttccaagaagaggaaggtaag
Rps10 199 bp downstream (Chr17:27,771,920-27,771,988)	gtggccttcaaactcctctc	actcagagtcgactgaagaaga
Rps24 0 bp upstream TSS (5'UTR) (Chr14:25,309,903-25,310,020)	cttgcgcgttgatatgattgg	gataagcgacggatagtgctg
Rpl7 141 bp downstream (Chr1:16,094,250-16,094,373)	ctcagtttgctcctggtactg	tgtatctgagtgtagcctgga
Rpl9 8 bp upstream (Chr5:65,782,562-65,782,678)	caaacagaggatgggttcagatt	gccctgacggattacaagaac
Rpl11 70 bp upstream (Chr4:135,609,214-135,609,356)	cggatggagacggatgaaag	ctcgtttgtctgcctagaagaa
Rpl13 18 bp upstream (Chr8:125,626,232-125,626,358)	cacttccctttcgcctgattt	ggcagagactcacctcctatac
Eef1g 197 bp downstream (Chr19:9,041,728-9,041,874)	gctccggtgattagggtcac	ctccaggccctagaaaccat
POMC 744 bp downstream (Chr12:3,953,603-3,955,695)	aggcagatggacgcacataggtaa	tccacttagaactggacagaggct
Ncl 479bp downstream (Chr1:88,255,445-88,255,551)	ctaaggttggccctctcttc	gatatcgagggttcggatgtag
Cdk7 47bp upstream (Chr13:101,500,944-101,501,053)	gtcctacggaagctgagttg	gtaacaaatggacactgactgc
Npas4 728bp downstream (Chr19:4,989,020-4,989,243)	ctatgcttgtggatcttggc	ctgaagccatcaccctttct
Mgmt 267bp upstream (Chr7:144,085,843-144,086,027)	gattcctagtgggcttaactcttc	ccagacctgaaactggttcttt

SUPPLEMENTAL FIGURES

Supplemental Figure S1. Supplemental cMYC and MYSM1 ChIP-qPCR analysis. The data demonstrates the binding of cMYC at known MYC target genes *Ncl* and *Cdk7*, the binding of MYSM1 at MYSM1 target genes *Npas4* and *Mgmt*, and the binding of both transcriptional regulators at *Rpl11*, consistent with previous data and confirming the specificity of the ChIP analyses. Data was acquired in Ba/F3 cells and is from one experiment. All Ct values were normalized to those of the pro-opiomelanocortin (*Pomc*) gene, which serves as a negative binding region. Enrichment was calculated relative to input DNA.

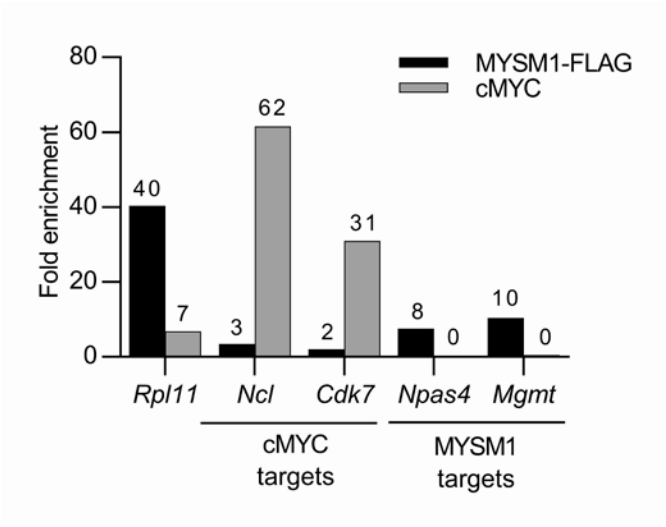

SUPPLEMENTAL REFERENCES

1. Adams JM, Harris AW, Pinkert CA, et al. The c-myc oncogene driven by immunoglobulin enhancers induces lymphoid malignancy in transgenic mice. Nature. 1985;318(6046):533-538.
2. Skarnes W, Rosen B, West A, et al. A conditional knockout resource for genome-wide analysis of mouse gene function. Nature. 2011;474(7351):337-342.
3. Nijnik A, Clare S, Hale C, et al. The critical role of histone H2A-deubiquitinase Mysm1 in hematopoiesis and lymphocyte differentiation. Blood. 2012;119(6):1370-1379.
4. Forster M, Belle JI, Petrov JC, Ryder EJ, Clare S, Nijnik A. Deubiquitinase MYSM1 Is Essential for Normal Fetal Liver Hematopoiesis and for the Maintenance of Hematopoietic Stem Cells in Adult Bone Marrow. Stem Cells Dev. 2015;24(16):1865-1877.
5. Belle JI, Petrov JC, Langlais D, et al. Repression of p53-target gene Bbc3/PUMA by MYSM1 is essential for the survival of hematopoietic multipotent progenitors and contributes to stem cell maintenance. Cell Death Differ. 2016;23(5):759-775.
6. Mills JR, Hippo Y, Robert F, et al. mTORC1 promotes survival through translational control of Mcl-1. Proc Natl Acad Sci U S A. 2008;105(31):10853-10858.
7. Mavrakis KJ, Wolfe AL, Oricchio E, et al. Genome-wide RNA-mediated interference screen identifies miR-19 targets in Notch-induced T-cell acute lymphoblastic leukaemia. Nat Cell Biol. 2010;12(4):372-379.
8. Belle JI, Langlais D, Petrov JC, et al. p53 mediates loss of hematopoietic stem cell function and lymphopenia in Mysm1 deficiency. Blood. 2015;125(15):2344-2348.
9. Langlais D, Couture C, Balsalobre A, Drouin J. The Stat3/GR interaction code: predictive value of direct/indirect DNA recruitment for transcription outcome. Mol Cell. 2012;47(1):38-49.
10. Wilson NK, Schoenfelder S, Hannah R, et al. Integrated genome-scale analysis of the transcriptional regulatory landscape in a blood stem/progenitor cell model. Blood. 2016;127(13):e12-23.
11. Wilson NK, Foster SD, Wang X, et al. Combinatorial transcriptional control in blood stem/progenitor cells: genome-wide analysis of ten major transcriptional regulators. Cell Stem Cell. 2010;7(4):532-544.
12. Belle JI, Wang H, Fiore A, et al. MYSM1 maintains ribosomal protein gene expression in hematopoietic stem cells to prevent hematopoietic dysfunction. JCI Insight. 2020;5(13):
13. Langmead B, Trapnell C, Pop M, Salzberg SL. Ultrafast and memory-efficient alignment of short DNA sequences to the human genome. Genome biology. 2009;10(3):R25.
14. Zhang Y, Liu T, Meyer CA, et al. Model-based analysis of ChIP-Seq (MACS). Genome Biol. 2008;9(9):R137.
15. Heinz S, Benner C, Spann N, et al. Simple combinations of lineage-determining transcription factors prime cis-regulatory elements required for macrophage and B cell identities. Mol Cell. 2010;38(4):576-589.
16. Thorvaldsdottir H, Robinson JT, Mesirov JP. Integrative Genomics Viewer (IGV): high-performance genomics data visualization and exploration. Brief Bioinform. 2013;14(2):178-192.
